# Exploring the concept of “inflammatory angiogenesis” in keratocystic odontogenic tumor

**DOI:** 10.4317/medoral.18351

**Published:** 2013-02-05

**Authors:** Mojgan Alaeddini, Esmat Mostafaloo, Omid Mirmohammadkhani, Nosratollah Eshghyar, Shahroo Etemad-Moghadam

**Affiliations:** 1 Assistant Professor, Dental Research Center, Tehran University of Medical Sciences, Tehran, Iran; 2 Dentist, Faculty of Dentistry, Qazvin University of Medical Sciences, Qazvin, Iran; 3 Oral and Maxillofacial Pathology Resident, Department of Oral and Maxillofacial Pathology, Faculty of Dentistry, Tehran University of Medical Sciences, Tehran, Iran; 4 Associate Professor, Department of Oral and Maxillofacial Pathology, Faculty of Dentistry, Tehran University of Medical Sciences, Tehran, Iran

## Abstract

Objective: The aim of the present study was to investigate the role of inflammation in angiogenesis of keratocystic odontogenic tumor (KCOT). Study Design: Twenty inflamed and 20 non-inflamed KCOTs were selected based on quantitative scoring of inflammation which was also applied on 20 radicular cysts. Microvessel density was assessed in all samples using CD34 antibody and angiogenesis was compared between the three groups. Statistical analysis was performed using one-way analysis of variance followed by post-hoc Scheffe test and P values less than 0.05 were considered significant. Results: A statistically significant difference in angiogenesis was found between radicular cysts and both inflamed and non-inflamed KCOTs (P < 0.001), but not between inflamed and non-inflamed KCOTs (P =0.347). Conclusion: Based on the results obtained in the present study, it seems that the effect of inflammation on angiogenesis in KCOT is minimal. However further investigation using other methods of evaluation is suggested to fully clarify the role of “inflammatory angiogenesis” in this neoplasm.

** Key words:**Keratocystic odontogenic tumor, radicular cyst, angiogenesis, inflammation.

## Introduction

Keratocystic odontogenic tumor (KCOT) is a cystic lesion derived from the dental lamina and differs from other odontogenic cysts in displaying an aggressive clinical behavior and repeated recurrences. These features along with its distinct histologic characteristics have been the basis of numerous investigations, leading to a change in nomenclature from odontogenic keratocyst to KCOT (1,2). Both the epithelial lining and stromal component have been proposed as being responsible for the uniqueness of this lesion (1,3,4).

Inflammation and angiogenesis are both stromal elements which have been considered as indications of host involvement in tumor maintenance, support and progression (5). Inflammation is the body’s reaction to any kind of assault including the presence of neoplastic tissues and is initiated by the immune system (6). Most neoplasms are generally known to secrete various cytokines and chemokines in order to recruit inflammatory cells which in turn assist tumor growth by secretion of proteolytic, angiogenic and mitogenic factors (7). KCOT has been reported to contain inflammation in its fibrous wall in 67.8% to 75% of cases (4,8) that can result from secondary infection from cortical bone perforations and periodontal ligament or other host-related causes apart from infectious agents (3). The effect of inflammation on the epithelial lining and connective tissue stroma of this tumor has been previously investigated and has shown changes in the morphology and proliferative activity of the epithelium and collagen structure of inflamed tumors and has even been proposed to complicate the diagnosis of this lesion (3,4,8-11). In addition numerous elements that can be secreted from inflammatory cells (e.g. TNF, IL-1α) have the ability to induce molecules such as keratinocyte growth factor and matrix metalloproteinases in the stroma of KCOT, leading to epithelial cell proliferation and possible influence on the expansion and behavior of the tumor resulting from bone resorption (10,12-14).

Angiogenesis involves the formation of new microvessels from preexisting vasculature and is an essential part of growth, whether in the form of normal tissues or neoplasms (15). Endothelial cells constitute the main core of this process and can be stimulated and recruited by both tumor- and inflammatory-cells, the latter being responsible for indirect neoplastic vascularization, a process termed “inflammatory angiogenesis” (6). Our previous work suggested a possible role for angiogenesis in KCOT (1), which has also been corroborated by others (16,17). Considering the presence of inflammation in a considerable number of reported KCOTs (3,4,8,10,11), we intended to explore the concept of “inflammatory angiogenesis” in this odontogenic tumor. Therefore immuno-histochemistry was used to compare inflammation and angiogenesis between KCOT and radicular cyst (RC) which is a common inflammatory cyst.

## Material and Methods

The protocol for this study was approved by the university Research and Ethics Committee. Archival haematoxylin/eosin-stained-slides of our departments were reviewed from 2000 to 2010 and after verification of the clinical and surgical data, 20 RCs and 60 KCOTs with various amounts of inflammation were chosen and their paraffin blocks retrieved. Presence of inflammation was based on a slight modification of the method described by Hirshberg et al. (3) and Kaplan and Hirshberg (4). The epithelial lining of the cysts demonstrated a length of at least 5mm attached to the underlying connective tissues, which in the KCOT samples had to have the typical parakeratinized lining required for correct diagnosis. All KCOTs were non-syndromic, primary (non-recurrent) and completely excised and none of them were preceded by decompression or marsupialization. Immunohistochemistry was performed on 5µm cut representative sections: dewaxing in xylene was followed by rehydration in graded alcohol and blocking endogenous peroxidase activity by treatment with hydrogen peroxide (3%) in phosphate buffered saline. Incubation with CD34 monoclonal antibody (QBEnd 10; Dako, Glostrup, Denmark) was performed (1:50 dilution, 30 min, room temperature); after antibody retrieval by placing sections treated with Tris buffer (10 mmol/l) and Ethylenediaminetetraacetic acid (1 mmol/l, PH=9.0) in a microwave oven (120°C) for 10 minutes. All sections were then incubated with secondary biotinylated antibody for 15 minutes followed by streptavidin for another 15 minutes. Color was developed with diaminobenzidine and finally the samples were counterstained with Mayer’s haematoxylin. Each section was accompanied by positive and negative controls, consisting of human tonsil and replacement of primary antibody with non-immune serum, respectively. When present, large vessels containing intraluminal red blood cells were used as internal positive control.

-Inflammation density and immunohistochemical scoring

Five consecutive high power fields (×400) were chosen from the connective tissue immediately adjacent to the basement membrane of the cystic epithelial lining and inflammatory cells were counted. Based on previous studies (3,4), the average of the separate numbers recorded for each field were scored as follows: a number of 0-15 cells were classified as non-inflammatory and the presence of more than 16 cells were considered as inflammatory KCOT. For assessment of angiogenesis, microvessel density (MVD) was calculated according to the method proposed by Weidner et al (18) using an Olympus BH2 microscope (Olympus, Tokyo, Japan) with a field size of approximately 0.18 mm2 at ×400 magnification. For each sample microvessels in three non-overlapping high power fields (×400) selected from vascular hotspots were counted and recorded as mean density. The mean number of microvessels per high power field was considered as MVD. Two pathologists simultaneously analyzed the microscopic slides using a double-headed microscope and disagreements were resolved through consensus and if needed a third observer was consulted.

-Statistical Analysis

One-way analysis of variance (ANOVA) followed by post-hoc Scheffe test was used for statistical analysis and P values less than 0.05 were regarded as significant.

## Results

**Figure 1:**
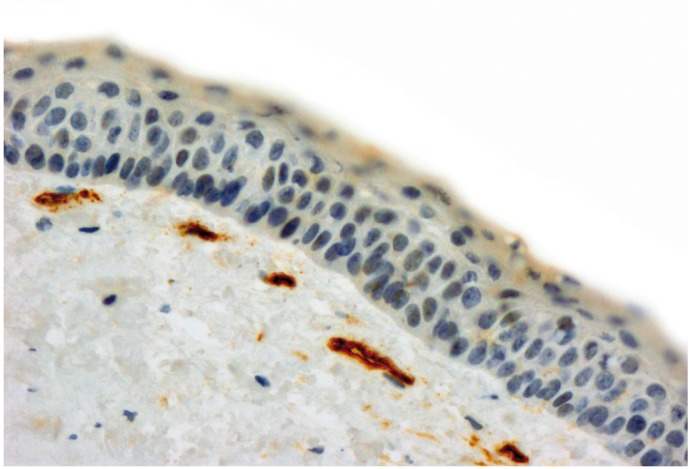
Microvessel density assessed by immunohistochemistry using monoclonal CD34 antibody in a representative noninflamed keratocystic odontogenic tumor (original magnification x400).

**Figure 2:**
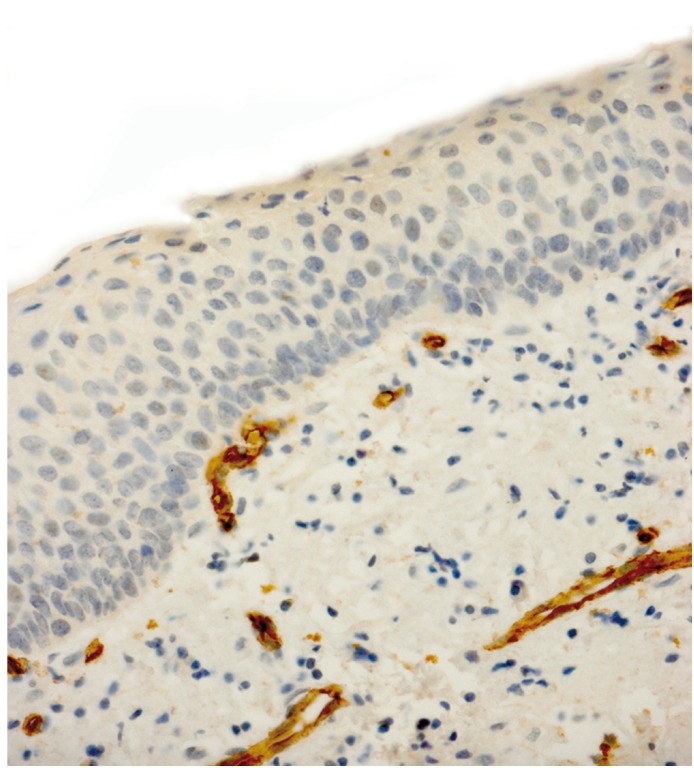
Microvessel density assessed by immunohistochemistry using monoclonal CD34 antibody in a representative inflamed keratocystic odontogenic tumor (original magnification x400).

**Figure 3:**
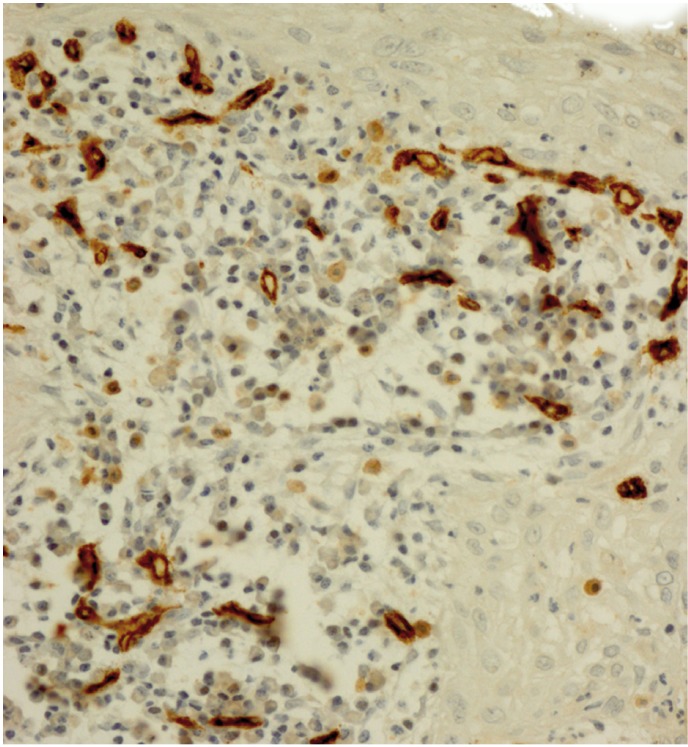
Microvessel density assessed by immunohistochemistry using monoclonal CD34 antibody in a representative radicular cyst (original magnification x400).

Our study sample consisted of 20 RCs along with 40 KCOTs, including 20 inflamed and 20 non-inflamed tumors which were chosen from the 60 initially selected cases based on the applied classification of inflammation. All endothelial cells of muscular-walled vessels showed immunostaining with CD34 antibody. In addition some inflammatory cells like mast cells and plasma cells were also positive for this antibody, which could be differentiated from individual endothelial cells by their morphologic features. Any spindle cell with or without finger-like projections was excluded during MVD counting. Mean microvessel density was 12.41 (range: 4.33-21.00) in non-inflammed KCOTs (Fig. 1), 16.17 (range: 8.33-43.00) in inflamed KCOTs (Fig. 2) and 27.40 (range: 10.00-47.30) in radicular cysts (Fig. 3) with the highest and lowest microvessels found in non-inflamed KCOTs and RCs, respectively ( Table 1).

**Table 1:**
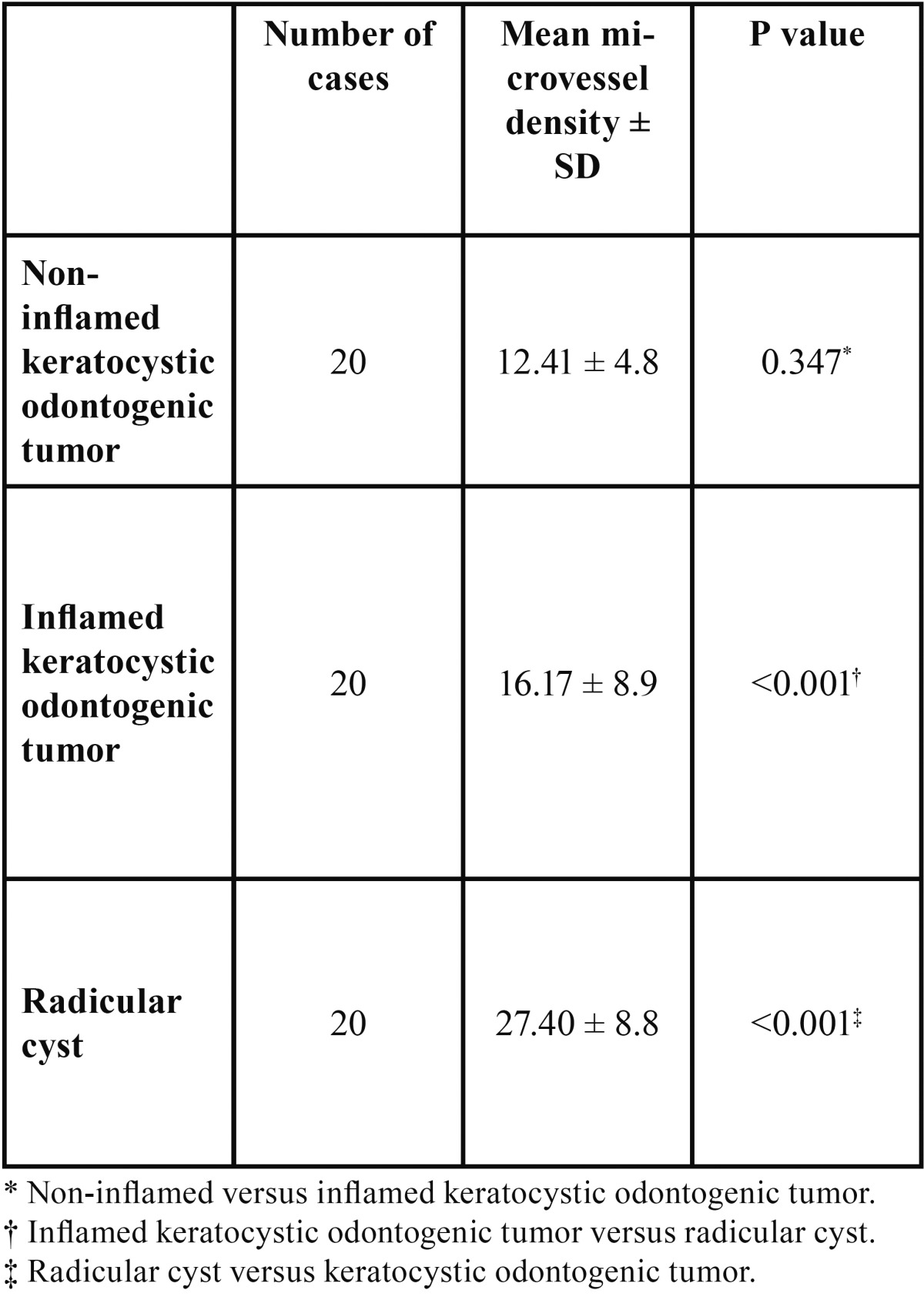
Comparison of angiogenesis between non-inflamed keratocystic odontogenic tumor, radicular cyst and inflamed keratocystic odontogenic tumor.

Comparison of the three groups by one-way analysis of variance revealed a statistically significant difference (P<0.001), while according to the post hoc test, we did not observe a significant difference in angiogenesis between the inflamed and non-inflamed KCOTs (P = 0.347), but the difference between radicular cysts and both inflamed (P < 0.001) and non-inflamed KCOTs (P < 0.001) was statistically significant.

In most of the inflamed KCOTs the epithelial lining demonstrated metaplasia to a non-keratinized squamous epithelium and thinning and even rete ridge formation were also observed in some specimens.

## Discussion

Angiogenesis or neovascularization ensues as a result of imbalance between pro- and anti-angiogenic factors, which can be secreted by the tumor itself or by other cells including those residing in the host connective tissue/neoplastic stroma or related to inflammation (6,19). Most studies have focused on the direct role of tumor cells in stimulating endothelial cells (6); however, molecules released by stromal cells can be equally potent in the induction or inhibition of angiogenesis. Muscle cells and platelets are known to release vascular endothelial growth factor (VEGF) (19) and inflammatory cells have been shown to demonstrate an indirect effect on angiogenesis by stimulating and recruiting endothelial cells required for neovascularization by a phenomenon termed “inflammatory angiogenesis” (6). The role of angiogenesis has been previously demonstrated in KCOT (1,16,17), in order to determine the possible mechanisms involved in this process we tried to clarify the contribution of inflammation in the formation of new blood vessels in this tumor. When comparing angiogenesis between inflamed and non-inflamed KCOTs, we did not find a significant difference, which may be due to the prevailing role of neoplastic cells in inducing angiogenesis. It seems that the inflammatory cells found in inflamed KCOTs, which have been reported to occur in 67.8%-75% of cases (4,8), are more likely to have secondarily penetrated into the tumor as opposed to being recruited for angiogenic purposes and can only minimally affect angiogenesis as shown by the non-significant increase in microvessel density in the present study. Mitrou et al. (20) have also reported that the expression of VEGF, a strong pro-angiogenic factor, was not dependant upon the presence of inflammation in KCOTs, which is in line with our findings. Hirshberg et al (3) have proposed the epithelial lining or resident fibroblasts as being a possible source for summoning inflammatory cells. In case this proves to be true, it seems that their main effect would be on features such as the collagen structure of inflamed tumors or epithelial proliferative activity (8,10) and cytokeratin expression (21), and they would only have a minor impact on the stromal vasculature.

The inflammation scoring system used in the current investigation was based on that described by Hirshberg et al. (3) who found a significant difference in the packing of stromal collagen fibers between KCOTs with low and high inflammation. Kaplan and Hirshberg (4) also showed a focal increase in Ki-67 labeling index of the epithelial linings in KCOTs which had moderate to severe inflammation in their underlying connective tissues. It may be probable that using further modifications in the applied scoring system or other evaluation techniques would change the results acquired in the current investigation. In addition it is noteworthy that our study was performed on a limited number of cases containing a specific number of inflammatory cells in the tumor stroma; maybe different results would be obtained if the inflammation score was higher in the inflamed KCOT group, leading to changes in the abovementioned hypothesis.

Inflammatory cells are generally considered to be related to angiogenesis in normal and lesional tissues. An increase in inflammatory cells can result in propagation of cytokines and chemokines, some of which are angiogenic (22). Microvessel count and MVD and have been found to be higher in cysts with more accentuated inflammation (20,23). Moreover, angiogenesis itself can upregulate inflammation through fascilitating and increasing transportation of inflammatory cells, providing oxygen and nutrients to the site harboring inflammation and finally increased secretion of inflammation attracting factors due to the existence of a higher number of endothelial cells in the region. The largest number of inflammatory cells found in inflamed KCOT in the present study was much less than the lowest number of cells observed in the radicular cysts. Therefore maybe the extremely high number of inflammatory cells in radicular cysts contributed to the significant difference found in the MVD score between the KCOTs and RCs evaluated in the current investigation. In RCs, there is a continuous secretion of bacterial toxins from the infected root canal which leads to a constant migration of inflammatory cells to the region (23,24). This is in contrast to the one-time entrance of inflammation to the fibrous wall of KCOTs following a single exposure through the cortical bone (3,4). Therefore the higher number of these cells would be expectable. Another contributing factor could be the difference in inflammatory cell types between these two lesions, which is composed mainly of lymphocytes in KCOTs compared to a more mixed population in RCs (24,25). Neutrophils and macrophages found in RCs are potent inducers of angiogenesis (6,24), which along with the other inflammatory cell types found in this lesion may have a stronger effect in producing new vasculature in comparison to the mere lymphocytic population of inflamed KCOTs.

In conclusion our data showed a significant difference in angiogenesis among RCs and inflamed and non-inflamed KCOTs and between RCs and both types of KCOTs, but not between inflamed and non-inflamed KCOTs; which suggest that angiogenesis may be an important factor in the development and biology of these lesions. However the exact role of inflammation in the angiogenic potential of KCOTs is still unclear and would need further investigation.
